# Evidence for Quinol Oxidation Activity of ImoA, a Novel NapC/NirT Family Protein from the Neutrophilic Fe(II)-Oxidizing Bacterium Sideroxydans lithotrophicus ES-1

**DOI:** 10.1128/mbio.02150-22

**Published:** 2022-09-15

**Authors:** Abhiney Jain, Anaísa Coelho, Joana Madjarov, Catarina M. Paquete, Jeffrey A. Gralnick

**Affiliations:** a BioTechnology Institute and Department of Plant and Microbial Biology, University of Minnesota — Twin Cities, St. Paul, Minnesota, USA; b Instituto de Tecnologia Química e Biológica António Xavier, Universidade Nova de Lisboa, Lisbon, Oeiras, Portugal; California Institute of Technology

**Keywords:** Fe(II)-oxidizing bacteria, quinol oxidoreductase, extracellular electron transfer, Mto, extracellular matrix electron transfer, iron oxidizing bacteria

## Abstract

*Sideroxydans* species are important chemolithoautotrophic Fe(II)-oxidizing bacteria in freshwater environments and play a role in biogeochemical cycling of multiple elements. Due to difficulties in laboratory cultivation and genetic intractability, the electron transport proteins required for the growth and survival of this organism remain understudied. In Sideroxydans lithotrophicus ES-1, it is proposed that the Mto pathway transfers electrons from extracellular Fe(II) oxidation across the periplasm to an inner membrane NapC/NirT family protein encoded by *Slit_2495* to reduce the quinone pool. Based on sequence similarity, Slit_2495 has been putatively called CymA, a NapC/NirT family protein which in Shewanella oneidensis MR-1 oxidizes the quinol pool during anaerobic respiration of a wide range of substrates. However, our phylogenetic analysis using the alignment of different NapC/NirT family proteins shows that Slit_2495 clusters closer to NirT sequences than to CymA. We propose the name ImoA (inner membrane oxidoreductase) for Slit_2495. Our data demonstrate that ImoA can oxidize quinol pools in the inner membrane and is able to functionally replace CymA in S. oneidensis. The ability of ImoA to oxidize quinol *in vivo* as opposed to its proposed function of reducing quinone raises questions about the directionality and/or reversibility of electron flow through the Mto pathway in S. lithotrophicus.

## OBSERVATION

The contribution of chemolithoautotrophic Fe(II)-oxidizing bacteria to iron cycling in the environment has been widely recognized in recent years (1). These bacteria rely on Fe(II) as the electron donor while reducing oxygen to fix carbon dioxide (1–4). Despite the relevance of these bacteria, the understanding of electron transfer proteins required for their growth and survival in the environment is primarily based on genomic analysis, heterologous expression, and biochemical analysis (5–9) due to their genetic intractability. Although a genetic system has been reported for the obligate Fe(II)-oxidizing bacterium Mariprofundus ferrooxydans (10), methods to generate gene deletions in this strain are still lacking, which is a critical tool for validating predictions of functionality.

In Gram-negative bacteria, Fe(II) oxidation would likely require either an outer membrane protein or a periplasmic protein with access to extracellular environment, e.g., through an outer membrane porin, to oxidize Fe(II) extracellularly (5). The electrons released from Fe(II) oxidation would then be transferred to quinone pools in the inner membrane via periplasmic and inner membrane electron transferring proteins (5). Based on genomic analysis, the *bc*_1_ complex (Complex III) has been proposed to be the inner membrane quinone reductase in most chemolithoautotrophic Fe(II)-oxidizing bacteria used to generate a proton gradient to drive NADH production (5). A similar function has also been proposed for a NapC/NirT family tetraheme containing protein encoded by *Slit_2495* during Fe(II) oxidation in Sideroxydans lithotrophicus ES-1 (9), a freshwater chemolithoautotroph (5–7). The proposed function of Slit_2495 is based on its sequence similarity with CymA, another NapC/NirT protein known to oxidize quinol during reduction of multiple electron acceptors in Shewanella oneidensis MR-1 (11). The S. lithotrophicus ES-1 genome also encodes a putative *bc*_1_ complex (7), making the possible quinone reductase role of Slit_2495 functionally redundant (8). Since S. lithotrophicus ES-1 is not yet genetically tractable, we heterologously expressed Slit_2495 and performed *in vivo* assays in a S. oneidensis mutant where CymA was replaced by Slit_2495. Our results revealed that Slit_2495 can functionally replace CymA in several modes of anaerobic respiration. Slit_2495 has been putatively called CymA in the literature (6, 8). However, phylogenetic analysis shows that Slit_2495 is more closely related to NirT from Pseudomonas than to CymA from *Shewanella*. Based on our analysis, we propose that *Slit_2495* be named *imoA* for inner membrane oxidoreductase.

**FIG 1:**
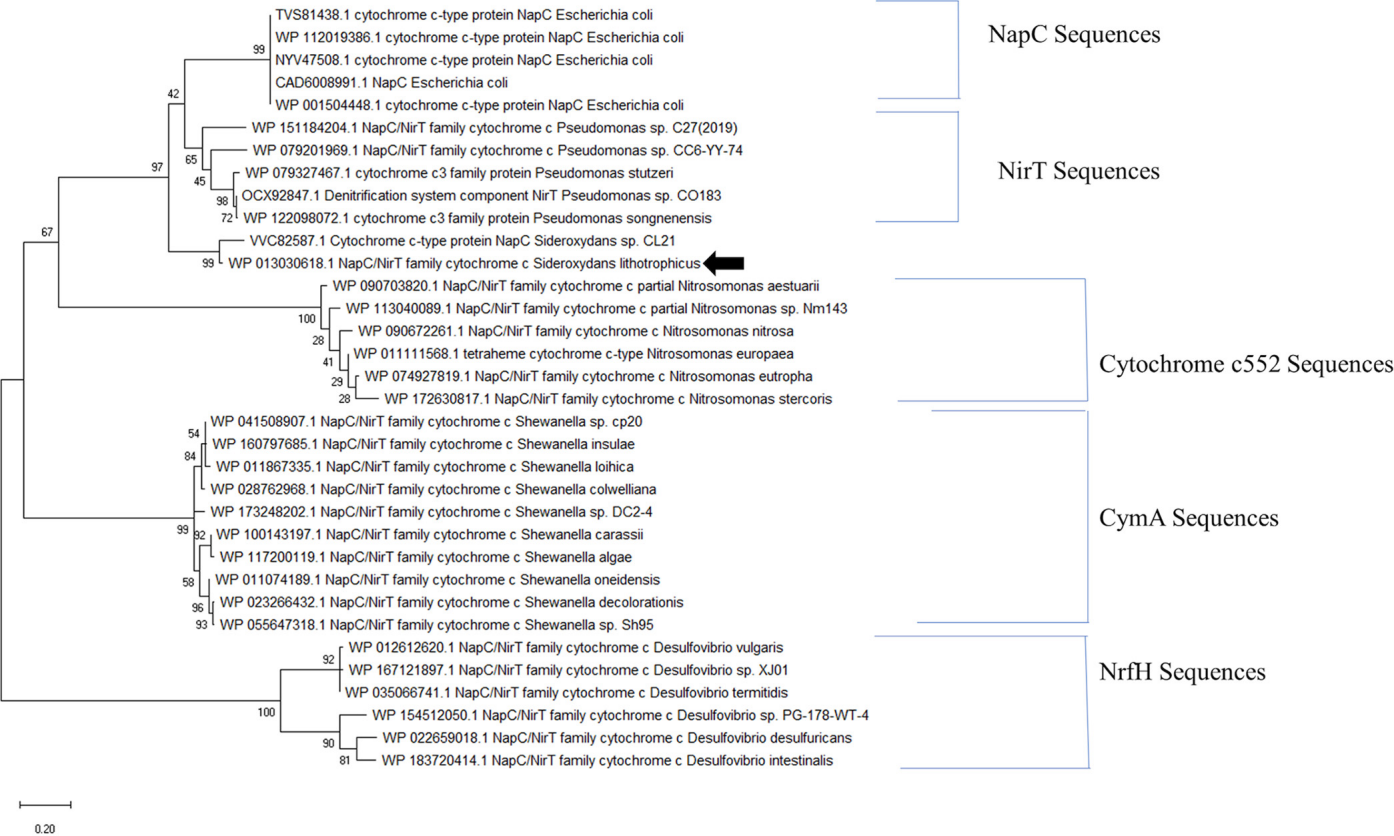
Phylogenetic analysis of ImoA with respect to other NapC/NirT proteins. Maximum likelihood tree was built using 2,000 bootstrap replications. Clusters of different NapC/NirT protein families used in the analysis are identified by the brackets labeled with specific proteins and black arrow points to ImoA sequence. The tree is drawn to scale, and the scale bar represents substitutions per site.

Phylogenetic analysis of ImoA was performed with respect to related NapC/NirT family proteins. We included sequences of CymA from *Shewanella* species, NirT from Pseudomonas species, NrfH from *Desulfovibrio* species, the membrane-bound *cytochrome c_552_* sequences from *Nitrosomonas* species along with NapC/NirT family protein sequences from 2 *Sideroxydans* species. Sequences from TorC or DorC were not included in the analysis since both proteins contain 5 heme binding sites compared to 4 heme binding sites in ImoA. Our phylogenetic analysis showed that ImoA clustered close to NapC sequences from Escherichia coli and NirT sequences from Pseudomonas (Fig. 1). Furthermore, the protein sequence of ImoA is 64% identical to NirT from Pseudomonas stutzeri and 54% identical to NapC from E. coli, whereas it is only 33% identical to CymA from S. oneidensis MR-1.

**FIG 2:**
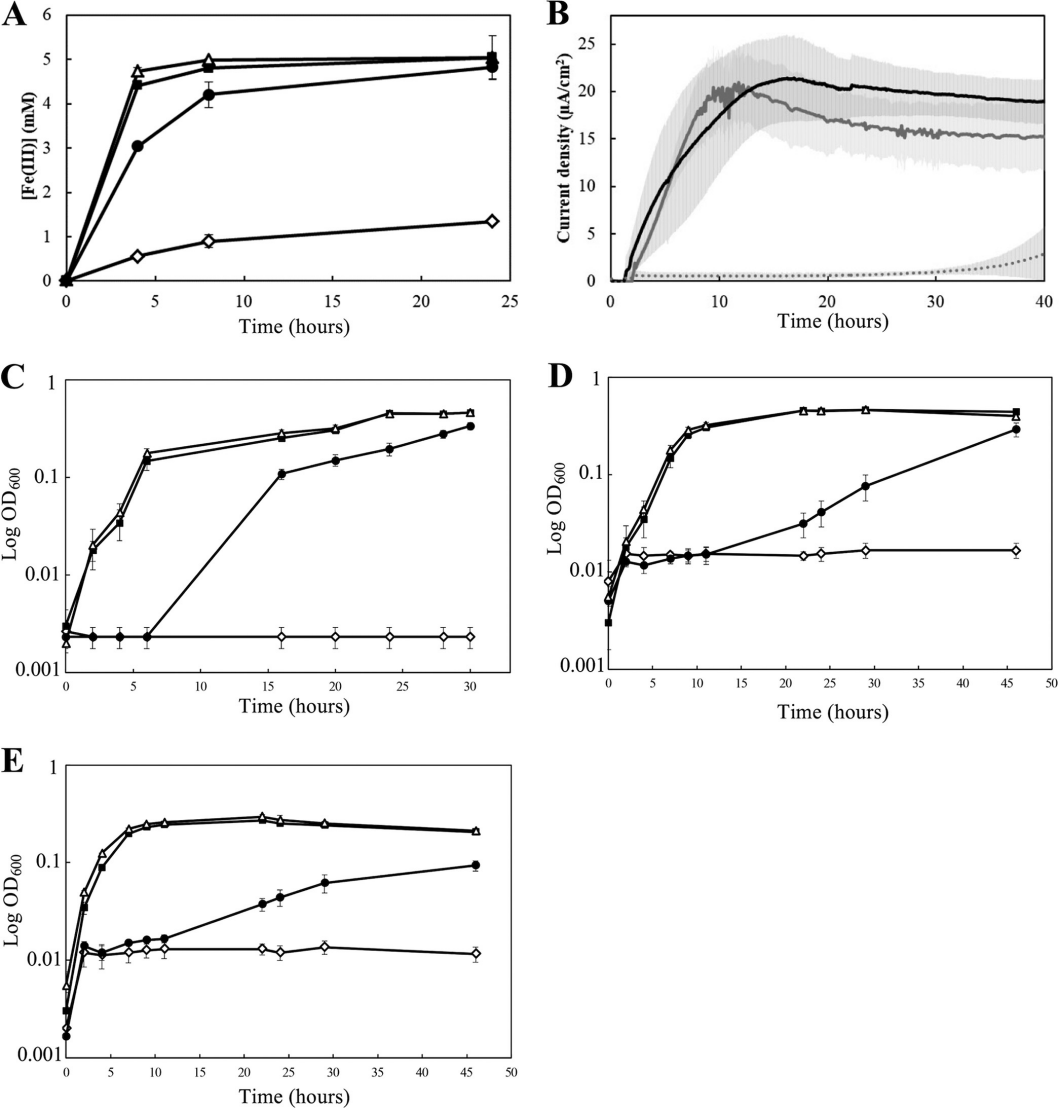
Functionality of ImoA to oxidize quinols in CymA-dependent respiratory pathways. (A) Resting cell Fe(III) citrate reduction: S. oneidensis containing pBBR1MCS-2 (Δ), Δ*cymA* containing pBBR1MCS-2 (◊), Δ*cymA* containing pBBR1MCS-2::*cymA* (■) and Δ*cymA* containing pBBR1MCS-2::*imoA* (●). Error bars represent standard deviation of the mean from experiments performed in triplicate. (B) Current density produced by the different Δ*cymA* strains containing pBBR1MCS-2::*imoA* (black line), pBBR1MCS-2::*cymA* (gray line), or empty pBBR1MCS-2 (gray dotted line) in a bioelectrochemical reactor. Error bars shown in gray represent standard deviation of the mean from experiments performed in two triplicates. ImoA can functionally replace CymA on growth with different electron acceptors including fumarate (C), DMSO (D) and nitrate (E) while using lactate as the electron donor. S. oneidensis containing pBBR1MCS-2 (Δ), Δ*cymA* containing the empty pBBR1MCS-2 vector (◊), Δ*cymA* containing pBBR1MCS-2::*imoA* (●) and Δ*cymA* containing pBBR1MCS-2::*cymA* (■). Error bars represent standard deviation of the mean from experiments performed in triplicate.

We tested the ability of ImoA to oxidize quinol pools in the inner membrane by expressing it in a S. oneidensis mutant lacking *cymA*. Expression of *imoA* complemented the Fe(III) citrate and anode reduction activities in S. oneidensis mutant lacking *cymA* (Fig. 2A and B), consistent with ImoA oxidizing quinols *in vivo*. Furthermore, expression of *imoA* partially complemented the growth defects of the *cymA* mutant strain with dimethyl sulfoxide (DMSO) (Fig. 2C), fumarate (Fig. 2D), and nitrate (Fig. 2E) as electron acceptors, consistent with the ability of ImoA to transfer electrons to multiple periplasmic redox proteins in S. oneidensis.

### IMPLICATIONS

In S. lithotrophicus ES-1 ImoA is a *c*-type tetraheme cytochrome of the NapC/NirT family proposed to act as a quinone reductase, receiving electrons from iron oxidizing extracellular electron uptake pathway to reduce the quinone pool (5–7). While ImoA may indeed mediate inward electron flow in S. lithotrophicus, another quinone reductase (a putative *bc*_1_ complex) is also encoded in the genome, and it is unclear what, if any, advantages would be gained by having 2 different quinone reductase systems. While ImoA was previously proposed to function as a quinone reductase during Fe(II) oxidation (5), the results presented in this work (Fig. 2) suggest the possibility that ImoA may oxidize quinol pools. However, S. lithotrophicus has been reported to be metabolically limited, and known to grow only using either Fe(II) or thiosulfate oxidation (7). The potential function of ImoA to oxidize quinol in S. lithotrophicus is intriguing from both physiological and ecological perspectives. Fe(II) oxidation pathways are dependent on electron transport chains that require quinone reduction rather than a quinol oxidation (5). If ImoA oxidizes quinol, as indicated by our data, what role could it be playing in S. lithotrophicus? By analyzing the S. lithotrophicus ES-1 genome, we identified genes that encode putative periplasmic redox proteins that are known to either directly or indirectly accept electrons from NapC/NirT family quinol oxidizing proteins, including *nirS* (Slit_1129) and several homologs of *nirM*. The protein sequence encoded by *Slit_1129* is 68% identical to NirS from P. aeruginosa, while NirM is known to transfer electrons from NirT to NirS during nitrite reduction (12). Interestingly, MtoD is homologous to NirM (28% identity and 43% similarity), suggesting that S. lithotrophicus may be able to reduce nitrite, though this capability has not been demonstrated (7). Our work suggests that S. lithotrophicus, and other ImoA-containing Fe(II)-oxidizing bacteria may be more metabolically versatile than initially observed. Importantly, heterologous protein expression can provide insight into the function of novel electron transfer pathways from challenging environmental microbes, complementing biochemical approaches and motivating development of native genetic methods.

All methods are described in the supplementary information (TEXT S1).

10.1128/mbio.02150-22.1 TEXT S1 Supplemental materials and methods. Download TEXT S1, PDF file, 0.1 MB. Copyright © 2022 Jain et al. 2022 Jain et al. https://creativecommons.org/licenses/by/4.0/ This content is distributed under the terms of the Creative Commons Attribution 4.0 International license.
