# Supplementary material for: Evidence for Quinol Oxidation Activity of ImoA, a Novel NapC/NirT Family Protein from the Neutrophilic Fe(II)-Oxidizing Bacterium Sideroxydans lithotrophicus ES-1
Source: mBio. 2022 Sep 15;13(5):e02150-22. doi: 10.1128/mbio.02150-22 (PMC9600427; doi:10.1128/mbio.02150-22)
Supplement: TEXT S1 [file mbio.02150-22-s0001.pdf]

Supplemental Information

Evidence for quinol oxidation activity of ImoA, a novel NapC/NirT family protein from the neutrophilic Fe(II)-oxidizing bacterium *Sideroxydans lithotrophicus* ES-1

Abhiney Jain[a], Anaísa Coelho[b], Joana Madjarov[b], Catarina M. Paquete[b] and Jeffrey A. Gralnick[a*]

* For manuscript correspondence: cpaquete@itqb.unl.pt or gralnick@umn.edu

Materials and Methods

Bacterial cultivation

Sideroxydans lithotrophicus ES-1 was obtained from the National Center for Marine Algae and Microbiota (https://ncma.bigelow.org) culture collection and was grown on modified Wolfe's mineral medium (MWMM) [1], buffered to pH 6.5 with MES buffer. The culture was grown in 60 mL serum bottles containing 20 mL of ASW medium with N_2:CO_2 (80:20) headspace, and sealed with butyl rubber stoppers. Sealed serum bottles containing the medium were autoclaved and added with 0.1 mL of filtered ferrous chloride solution (100 mM), and 1 mL of filtered air to introduce oxygen as electron acceptor. 0.1 mL of filtered ferrous chloride solution (100 mM), and 1 mL of filtered air were added at every 24 hours to the serum bottles. *Shewanella oneidensis* strains were grown in lysogeny broth (LB) medium. 50 µM kanamycin was added to the medium when required.

S. oneidensis strain construction

Genomic DNA *of S. lithotrophicus* ES-1 was extracted using Qiagen DNeasy PowerSoil kit. The gene encoding Slit_2495 was amplified from *S. lithotrophicus* ES-1 genomic DNA using the

primers, NNNNNNCTCGAGATGAATAACAAGACTGGCATCCTGAAA and NNNNNNACTAGTTCACTCCTCATCCGGATCG. The amplified fragment was cloned into the pBBR1MCS-2 expression vector [2], which was transformed into chemically competent *E. coli* WM3064 cells [3] and selected on LB medium plates containing 50 µM kanamycin and 360 µM diaminopimelic acid (DAP) and used to conjugate the plasmid into Δ*cymA S. oneidensis* [4]. *S. oneidensis* cells containing the plasmid were selected on LB plates containing 50 µM kanamycin without DAP.

Fe(III) citrate reduction assay

Fe(III) citrate reduction assays were performed as described in [5]. Briefly, *S. oneidensis* cells were freshly struck from -80 °C glycerol stocks to LB plates containing 50 µM kanamycin. Tubes containing LB liquid medium with 50 µM kanamycin were inoculated with single colonies and incubated aerobically in a shaker at 30 °C. The cells were washed with *Shewanella* basal medium (SBM) [6] and resuspended in the same medium to obtain a cell density of 10^9 cells/mL. 30 µL of the resuspended cells were added to 270 µL of SBM containing 20 mM sodium lactate and 5 mM of Fe(III) citrate in a 96 well plate. The 96 well plate was placed inside a sealable chamber which was made anaerobic by flushing with oxygen free argon and incubated at 30 °C. Samples were collected periodically to quantify Fe(II), produced as a result of Fe(III) reduction, using the ferrozine assay [7].

Growth on anodes

S. oneidensis strains containing the desired plasmids were grown in three-electrode bioelectrochemical reactors. The reactors were single chambered and made out of 100 mL Schott glasses closed with butyl rubbers and fixed by screw caps to guarantee anaerobic conditions during the experiment. The working anode was made of graphite felt (GFD 2.5 from Sigracell (Germany), round size of 13 mm diameter) and stamped into a self-made electrode holder (hungate screw cap) with a silicon stopper. The counter electrode was a graphite rod and both electrodes were connected with titanium wires. The Ag/AgCl (3 M KCl) reference electrode (IJ Cambria) and working and counter electrode were inserted through a hole previously drilled into

the rubber. The surface of the anode electrode in contact with the medium was of 9 mm diameter (= 0.64 cm^2). Prior to autoclaving, the working electrode was immersed in isopropanol and washed with deionized water. Before use, the reactors were filled with deionized water and autoclaved. The bacteria were grown overnight in oxic LB medium containing 50 µM kanamycin, washed with SBM and inoculated in anoxic SBM containing 20 mM sodium lactate in the reactors. All the experiments were performed in triplicate at 30 ºC using a Dropsens multipotentiostat in the chronoamperometric mode applying 200 mV vs. Ag/AgCl and measuring the current every 30 seconds. Approximately 2 hours after the experiment started, washed *S. oneidensis* cells were added to the reactors with a resulting OD_{600nm} of 1.0.

Growth curves

S. oneidensis strains containing the desired plasmid were freshly struck from -80 °C glycerol stocks to LB plates with 50 µM kanamycin. Tubes containing LB liquid medium with 50 µM kanamycin were inoculated with single colonies and incubated aerobically while shaking at 30 °C. Overnight cultures were washed with SBM and inoculated into anoxic SBM containing 20 mM sodium lactate and 40 mM of the respective electron acceptors. Growth of different strains was measured over time using optical density (OD_{600nm}).

Phylogenetic analysis

Amino acid sequences representing CymA, NirT, NrfH , cytochrome c552 and NapC/NirT proteins from *S. lithotrophicus* ES-1 and *Sideroxydans spp.* CL21 were downloaded from NCBI (https://www.ncbi.nlm.nih.gov/protein/) and aligned using ClustalW [8]. MEGA7 was used to generate a phylogenetic tree using the maximum likelihood method with 2,000 bootstrap replications, based on the JTT matrix-based model [9, 10].

References

1. Emerson D, Moyer C. Isolation and characterization of novel iron-oxidizing bacteria that grow at circumneutral pH. Appl Environ Microbiol. 1997;63(12):4784-4792.

2. Kovach ME, Elzer PH, Hill DS, Robertson GT, Farris MA, Roop II RM, Peterson KM. Four new derivatives of the broad-host-range cloning vector pBBR1MCS, carrying different antibiotic-resistance cassettes. Gene. 1995;166(1):175-176.
3. Saltikov CW, Newman DK. Genetic identification of a respiratory arsenate reductase. Proc Natl Acad Sci USA. 2003;100(19):10983-10988.
4. Ross DE, Flynn JM, Baron DB, Gralnick JA, Bond DR. Towards electrosynthesis in *Shewanella*: energetics of reversing the Mtr pathway for reductive metabolism. PLoS One. 2011;6:e16649.
5. West EA, Jain A, Gralnick, JA. Engineering a native inducible expression system in *Shewanella oneidensis* to control extracellular electron transfer. ACS Syn Biol. 2017:6(9):1627-1634.
6. Hau HH, Gilbert A, Coursolle D, Gralnick, JA. Mechanism and consequences of anaerobic respiration of cobalt by *Shewanella oneidensis* strain MR-1. Appl. Environ. Microbiol. 2008; 74:6880-6886.
7. Stookey LL. Ferrozine—a new spectrophotometric reagent for iron. Anal. Chem. 1970; 42:779−781.
8. Thompson JD, Higgins DG, Gibson TJ. CLUSTAL W: improving the sensitivity of progressive multiple sequence alignment through sequence weighting, position-specific gap penalties and weight matrix choice. Nucleic Acids Res. 1994;22:4673– 4680.
9. Jones DT, Taylor WR, Thornton JM. The rapid generation of mutation data matrices from protein sequences. Comput Appl Biosci. 1992;8:275–282.
10. Kumar S, Stecher G, Tamura K. MEGA7: Molecular Evolutionary Genetics Analysis version 7.0 for bigger datasets. Mol Biol Evol. 2016;33:1870 –1874.
